# Can UVA-light-activated riboflavin-induced collagen crosslinking be transferred from ophthalmology to spine surgery? A feasibility study on bovine intervertebral disc

**DOI:** 10.1371/journal.pone.0252672

**Published:** 2021-06-03

**Authors:** Ioannis Vasilikos, Graciosa Q. Teixeira, Andreas Seitz, Julia Nothelfer, Julian Haas, Hans-Joachim Wilke, Boris Mizaikoff, Jürgen Beck, Ulrich Hubbe, Cornelia Neidlinger-Wilke

**Affiliations:** 1 Department of Neurosurgery, Faculty of Medicine, Medical Center—University of Freiburg, University of Freiburg, Freiburg, Germany; 2 Laboratory of Experimental Neurosurgery (LENS), Medical Center, Faculty of Medicine, University of Freiburg, Freiburg, Germany; 3 Institute of Orthopaedic Research and Biomechanics, Trauma Research Center, University of Ulm, Ulm, Germany; 4 Institute of Analytical and Bioanalytical Chemistry, University of Ulm, Ulm, Germany; Massachusetts Eye & Ear Infirmary, Harvard Medical School, UNITED STATES

## Abstract

**Background:**

Collagen cross-links contribute to the mechanical resilience of the intervertebral disc (IVD). UVA-light-activated riboflavin-induced collagen crosslinking (UVA-CXL) is a well-established and effective ophthalmological intervention that increases the mechanical rigidity of the collagen-rich corneal matrix in Keratoconus. This study explores the feasibility, safety and efficacy of translating this intervention in reinforcing the IVD.

**Methods:**

Annulus fibrosus (AF) cells were isolated from bovine IVDs and treated with different combinations of riboflavin (RF) concentrations (0.05–8 mM) and UVA light intensities (0.3–4 mW/cm^2^). Metabolic activity (resazurin assay), cell viability (TUNEL assay), and gene expression of apoptosis regulators *C-FOS* and *PT5* were assessed immediately and 24 hours after treatment. Biomechanical effects of UVA-CXL on IVDs were measured by indentation analysis of changes in the instantaneous modulus and by peel-force delamination strength analysis of the AF prior and after treatment.

**Results:**

Different intensities of UVA did not impair the metabolic activity of AF cells. However, RF affected metabolic activity (p < 0.001). *PT53* expression was similar in all RF conditions tested while *C-FOS* expression decreased 24 hours after treatment. Twenty-four hours after treatment, no apoptotic cells were observed in any condition tested. Biomechanical characterizations showed a significant increase in the annular peel strength of the UVA-CXL group, when compared to controls of UVA and RF alone (p < 0.05). UVA-CXL treated IVDs showed up to 152% higher (p < 0.001) instantaneous modulus values compared to the untreated control.

**Conclusion:**

This is the first study on UVA-CXL treatment of IVD. It induced significantly increased delamination strength and instantaneous modulus indentation values in intact IVD samples in a structure–function relationship. RF concentrations and UVA intensities utilized in ophthalmological clinical protocols were well tolerated by the AF cells. Our findings suggest that UVA-CXL may be a promising tool to reinforce the IVD matrix.

## Introduction

Back pain is the most important single cause of disability worldwide, preventing patients from engaging in work and other everyday activities [1–3]. It is mostly associated with pathologies of the intervertebral disc (IVD).

The IVD is the principal joint between two vertebrae in the spinal column and provides stability while facilitating multiaxial motions [4]. Its unique structure consists of two domains, the annulus fibrosus (AF) with concentric organized collagen-rich lamellas, surrounding a gelatinous nucleus pulposus (NP) with randomly organized collagens embedded in a highly hydrated aggrecan-rich matrix [4, 5]. IVD’s mechanical integrity depends mostly on cross-links among collagen fibers, proteoglycans and elastin present in the extracellular matrix [6, 7]. Age-related changes in the disc structure, associated with an overtime-accumulated stress due to biomechanical overload, can result in disc degeneration and damage of those interlinks leading to increased matrix turnover, changes in the cross-link profile of collagen fibers and decreased endogenous collagen cross-linking [6, 7]. Additionally, progressive aggrecan degradation leads to a reduced water-binding capacity [5, 6]. These alterations cause debilitation of tissue strength and loss of mechanical properties, thereby contributing to biomechanical failure, disc herniation, instability and pain [8].

Exogenous cross-linking of IVD collagen fibers seems to be a promising tool. Biomechanical experiments with bovine IVD have shown that exogenous collagen cross-linking with genipin (a natural cross-linking agent) caused a higher resilience under compressive creep loading along with a decrease in both tensile stresses and strains [9, 10]. UV-light-activated riboflavin-induced collagen crosslinking (UVA-CXL) is a well-established safe and effective intervention in ophthalmology that reinforces ectatic corneas [11–14]. The cornea is initially saturated with the photosensitizer riboflavin (RF, Vitamin B2, usually between 0.1 and 0.3%), which penetrates the collagen-rich stroma. In a second step, UVA light (365–375 nm) activates RF producing singlet oxygen (^1^O_2_) molecules, that catalyze carbonyl-based crosslinking reactions among collagen fibrils [15]. This process increases the biomechanical strength of the tissue up to 350% [16].

A plethora of ophthalmological protocols has been suggested combining different UVA and RF parameters [17]. Most importantly, the biomechanical effects of UVA-CXL on ectatic corneas have been shown to be stable 10 years after the intervention [11].

The successful application of UVA-CXL in ophthalmology, and the collagen rich nature of both corneas and IVDs, led us to hypothesize that this intervention could be translated in spinal surgery, where a reinforcement of the weakened or damaged IVD matrix would be a promising treatment option for disc degeneration-associated clinical problems.

This feasibility and safety study had two goals:

1. To analyze the effects of various RF, UVA or combined parameters on the metabolic activity and gene expression of isolated AF cells
2. To assess the influence of UVA-CXL on the biomechanical properties of bovine IVDs.

## Materials and methods

### Tissue dissection and cell isolation

Intervertebral discs (caudal levels C2–3 to C6–7) were isolated from bovine tails (age: 18–24 months-old), within 3 hours postmortem (Fleischmarkt Donautal, Ulm), as previously described [18]. Briefly, muscles and ligaments were removed and the mid-region of each IVD was isolated from the vertebral bodies using a custom-made standardized guillotine. Annulus fibrosus (AF) tissue rings were prepared and either used for cell isolation or directly for UVA-light activated riboflavin-induced crosslinking (UVA-CXL).

For cell isolation, the AF tissue was dissected into 2–3 mm^3^ fragments and enzymatically digested overnight in 0.8 mg/mL collagenase type I (Sigma-Aldrich, St. Louis, MO, USA) in IVD medium bovine serum-free, containing low-glucose Dulbecco’s Modified Eagle Medium (DMEM), with 1% Penicillin-Streptomycin (10000 U/mL), 0.5% Amphotericin B (250 μg/mL), 1% non-essential amino acids (all from Gibco, Waltham, MAs, EUA) and 1.5% of a 5 M NaCl/0.4 M KCl solution (to adjust osmolarity to 400 mOsm), under agitation (100 rpm), 37°C, reduced oxygen atmosphere (6% O_2_ and 8.5% CO_2_) and saturated humidity. The cell suspension was filtered through a 70-μm filter (BD Falcon, Franklin Lakes, NJ, USA) to remove tissue debris and centrifuged at 300g for 10 min. AF cells were seeded at a density of 3000 cells/cm^2^ in IVD medium supplemented with 5% fetal bovine serum (FBS, Gibco). The cells were expanded at 37°C, reduced oxygen atmosphere (6% O_2_ and 8.5% CO_2_) and saturated humidity. Medium was exchanged twice a week and cells were trypsinized when 70% confluency was reached. AF cells from 2 donors were used in passage 6–8. Experiments were repeated in duplicate or triplicate (n = 4–6).

### Riboflavin (RF) and UVA-light treatment of AF cells in 2D culture

**Fig 1:**
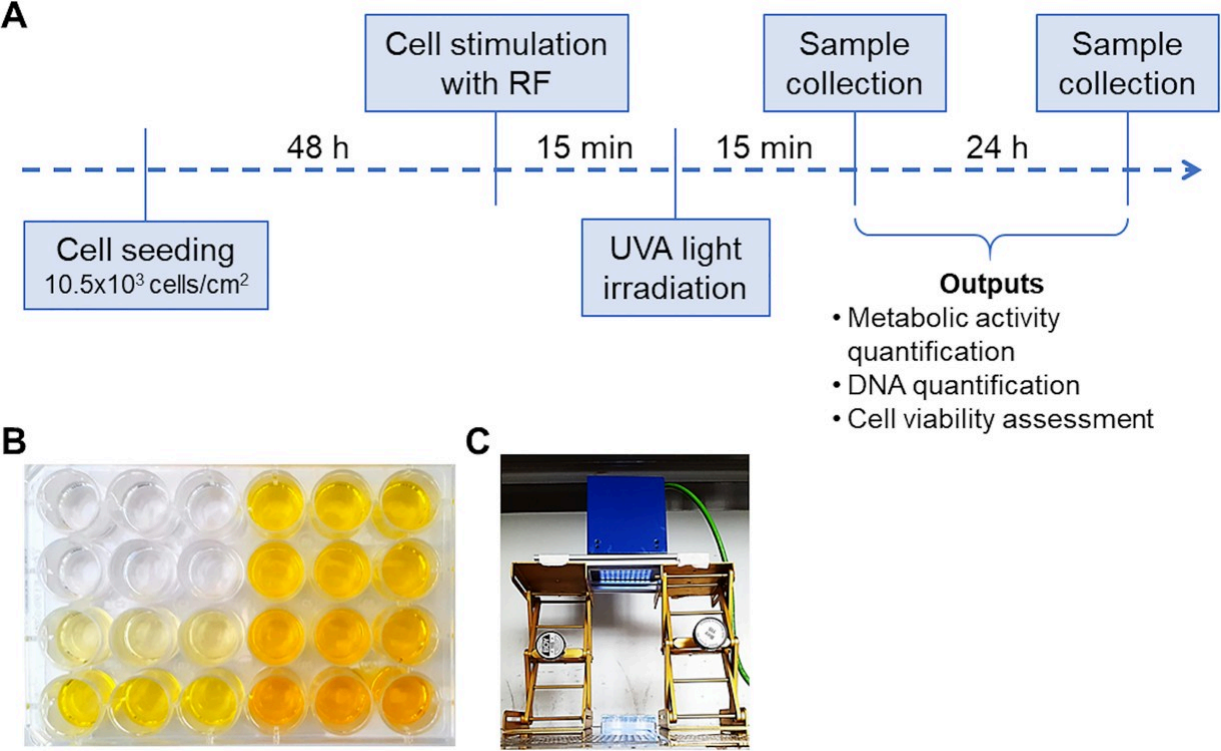
Annulus fibrosus (AF) cell treatment with different concentrations of Riboflavin (RF, 0.05, 0.5, 2 and 8 mM) and UVA light intensities (0.3, 3 and 4 mW/cm^2^). A) Experimental timeline. B) AF cells treated with RF. C) Experimental setup for AF cells exposure to UVA light.

AF-isolated cells were seeded in tissue culture plates (Corning, New York, NY, USA) at a density of 10.5x10^3^ cells/cm^2^ and left to adhere for 48 hours (Fig 1A). Afterward, cells were treated with 0.05, 0.5, 2 and 8 mM RF in phenol red-free DMEM/F12 (Gibco) for 15 min (Fig 1B), washed with Dulbecco’s phosphate-buffered saline (DPBS, Gibco) solution and kept in phenol red-free DMEM/F12 medium during UVA light irradiation for 15 min (Fig 1C). UVA irradiation was performed using an UVA-LED module (Opsytec Dr. Gröbel GmbH, Ettlingen, Germany). The UVA-LED module produced a homogenous field of UVA light at a wavelength of 365–370 nm. UVA-light intensity was adjusted by placing the UVA-LED module at different distances from the cell culture plate, based on pre-experiments. UVA-absorbance of the culture plates was also considered. This approach enabled cell irradiations with 0.3, 3.0 and 4.0 mW/cm^2^. The different intensities were calibrated using a radiometer (RM-12, Opsytec Dr. Gröbel). Untreated cells were used as controls.

AF cells were either analyzed immediately after UVA light irradiation or further kept in IVD medium for 24 hours. The mitochondrial metabolic activity was assessed immediately and 24 hours after the treatment. Afterward, AF cells were either kept in RLT buffer (Quiagen, Venlo, Netherlands) for gene expression analysis of metabolism-related markers or fixed in 4% buffered formaldehyde (Otto Fischar, Saarbrücken, Germany) solution.

### Mitochondrial metabolic activity of AF cells

Cell mitochondrial metabolic activity was accessed using the resazurin assay. Resazurin (Sigma-Aldrich) solution was added to IVD medium at a final concentration of 0.02 mg/ml. Samples were incubated for 2 hours at 37°C. Resazurin, which is a non-fluorescent reagent, when reduced by metabolically active cells is converted into the highly fluorescent product resofurin. Resofurin fluorescence intensity was measured in a spectrophotometer microplate reader (Tecan), with 530 nm excitation filters and 590 nm emission filters. Measurements were performed in triplicate and the blank control was included in each plate. Additionally, for each measurement, resorufin optical density was normalized to DNA content quantified with the Quant-iT™ PicoGreen® dsDNA Assay kit (Invitrogen, Carlsbad, CA, USA) per manufacturer instructions. Before DNA quantification, cells were lysed in 0.5 mg/ml Proteinase K (Sigma-Aldrich) solution (30 mM Tris-HCl, pH 8.0) for 1 hour at 37°C and then overnight at 56°C.

### RNA isolation and gene expression analysis

Total RNA was isolated and purified using the RNeasy Mini Kit and RNase-Free DNase (Quiagen) as per manufacturer instructions. RNA concentration and quality were determined by spectrophotometry (Spark, Tecan, Männedorf, Switzerland). The RNA was directly used for gene expression analysis. A one-step quantitative polymerase chain reaction (qPCR) was performed, including the cDNA synthesis and subsequent qPCR detection. For this, 1 μg of RNA was mixed with the SensiFAST™ SYBR® Hi-ROX One-Step Kit’s Mastermix (Bioline, London, UK) and specific bovine primers listed in Table 1 (ThermoFisher Scientific, Waltham, MA, USA). The reaction mixture was denatured at 95°C for 10 min, followed by 40 cycles of 95°C for 15 s and 60°C for 1 min. Relative expression of target genes was calculated according to the 2^-ΔCt^ method (ΔCt = Ct_(gene of interest)_−Ct_(*GAPDH*)_), according to published guidelines [19].

**Table 1:** Bovine primers.

Gene	Sequence (forward and reverse primers)	Product length (bp)
*C-FOS*	5’-CGG CTT TGC AGA CAG AGA TT-3’ 5’-CCC CCA CTC AGA TCA AGA GA-3’	148
*GAPDH*	5’-ACC CAG AAG ACT GTG GAT GG-3’ 5’-CAA CAG ACA CGT TGG GAG TG-3’	66
*TP53*	5’-ATT TAC GCG CGG AGT ATT TG-3’ 3’-CCA GTG TGA TGA TGG TGA GG-3’	174

bp, base pairs; GAPDH, glyceraldehyde 3-phosphate dehydrogenase; TP, tumour protein.

### Apoptosis of AF cells

**Fig 2:**
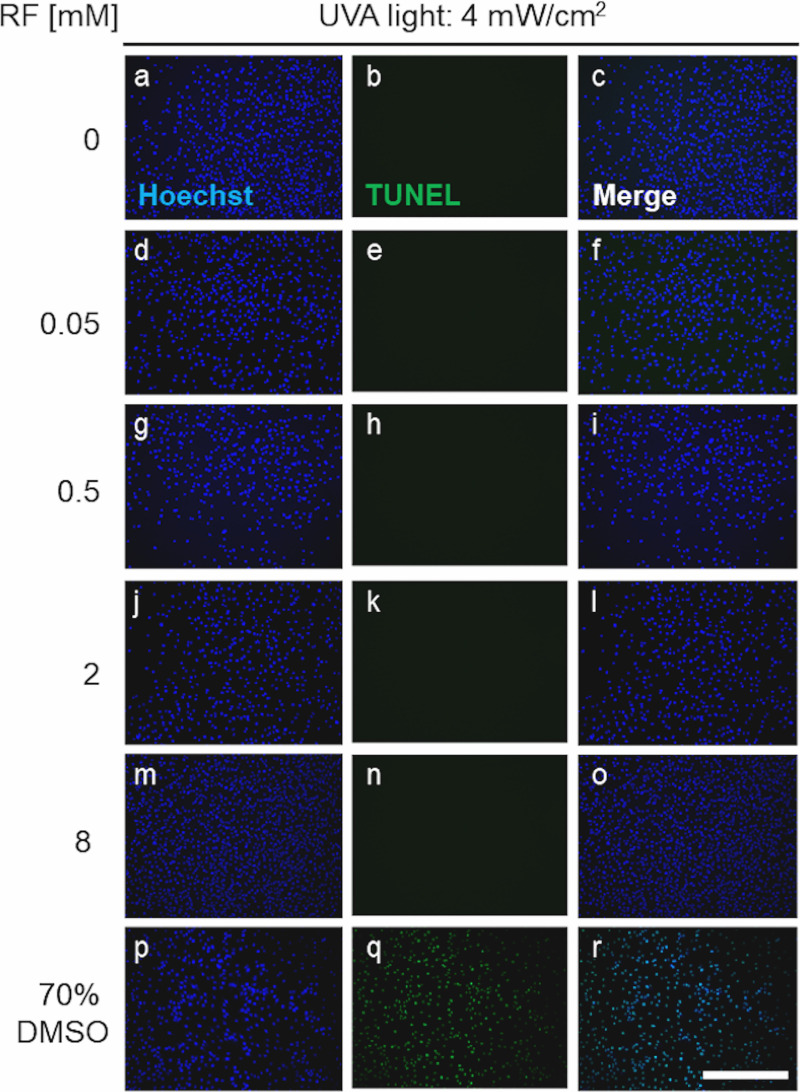
Representative fluorescence microscopy images of TUNEL apoptosis staining of AF cells, 24 hours after treatment with different riboflavin (RF) concentrations: 0.05, 0.5, 2 and 8 mM, exposed to 4 mW/cm^2^ UVA light. In parallel, cells were also kept in 75% DMSO in DMEM (positive control for apoptosis) or DMEM alone (negative control). Apoptotic cells are stained green (CF®488A) and cell nuclei is stained blue (Hoechst). Scale bar, 500 μm.

Twenty-four hours after RF and UVA light treatment, AF cell apoptosis was assessed using the CF^TM^ 488A TUNEL apoptosis detection kit (Biotium, Fremont, CA, USA) according to the manufacturer’s instructions. A group of AF cells treated with 70% dimethylsulfoxid (DMSO, Merk, Darmstadt, Germany) for 15 minutes was used as a positive control for cell apoptosis. Prior to the TUNEL staining, AF cells from all groups were fixed with 2% paraformaldehyde for 15 minutes. DNA was counterstained with 1 ng/mL Hoechst 33258 staining solution (Polysciences, Warrington, PA, USA) for 1 minute. Representative images were collected from randomly selected regions of interest using fluorescence microscopy (Leica DMI6000B, Leica Microsystems, Wetzlar, Germany). Apoptotic cells were stained green, while cell nuclei were stained blue (Fig 2).

### Delamination strength of fresh AF tissue

**Fig 3:**
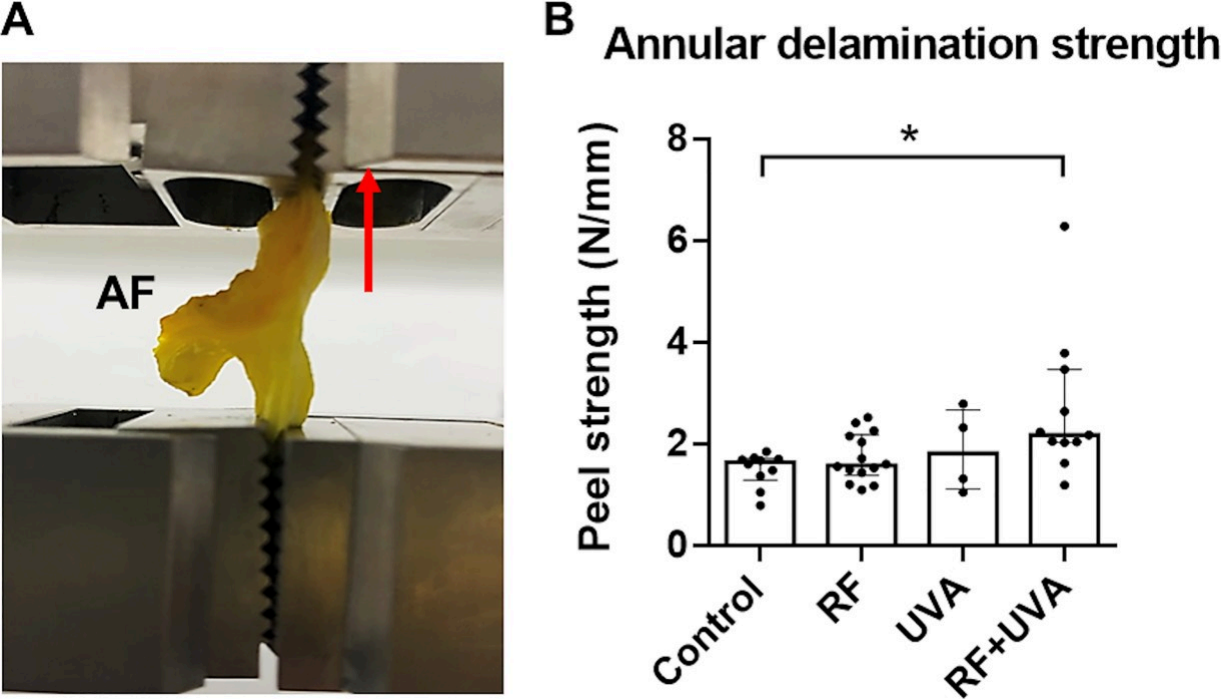
Annular delamination. A) Detailed look on the AF segment being pulled apart (at 0.5 mm/s; arrow) into a ‘Y’ configuration along a lamella boundary. B) Annular delamination strength as a function of the displacement rate (N/mm) for all conditions tested. AF tissue segments were i) submerged in 8 mM RF, ii) exposed to 3.5 mW/cm^2^ UVA light (UVA) or iii) submerged in RF followed by UVA light exposure (RF+UVA). Untreated AF tissues were kept in phenol red-free DMEM and analyzed as controls. Directly after treatment, the AF segments were pulled apart (at 0.5 mm/s) into a ‘Y’ configuration along a lamella boundary. Data is shown in scatter plots with bars as mean ± SD (n = 4–14 half AF rings from 20 IVDs isolated from 4 tails). Kruskal-Wallis test was used for statistical analysis, *P<0.05.

A subgroup of AF tissue was treated with 8 mM RF for 15 min, another with UVA light alone (3.5 mW/cm^2^) also for 15 min and a third subgroup with UVA-CXL. Untreated samples were used as controls. A total of 20 discs were isolated from 4 different tail specimens and each half AF ring was randomly attributed to a different group. The strength of the annulus matrix was evaluated using a peel test based on previously established methods [18, 20]. Briefly, a rectangular AF segment was dissected into a ‘Y’ configuration along a central lamella boundary and clamped into a ‘T’ configuration in a uniaxial material testing machine. The tissue was pulled apart along the lamellae at a constant speed of 0.5 mm/s, until complete separation of the sample (Fig 3A). The average force (N) in each force-displacement curve was divided by the width of the delaminated segment (mm) to calculate the delamination strength (N/mm) (Fig 3B).

### Biomechanical indentation analysis prior and after UVA-CXL on bovine IVDs

**Fig 4:**
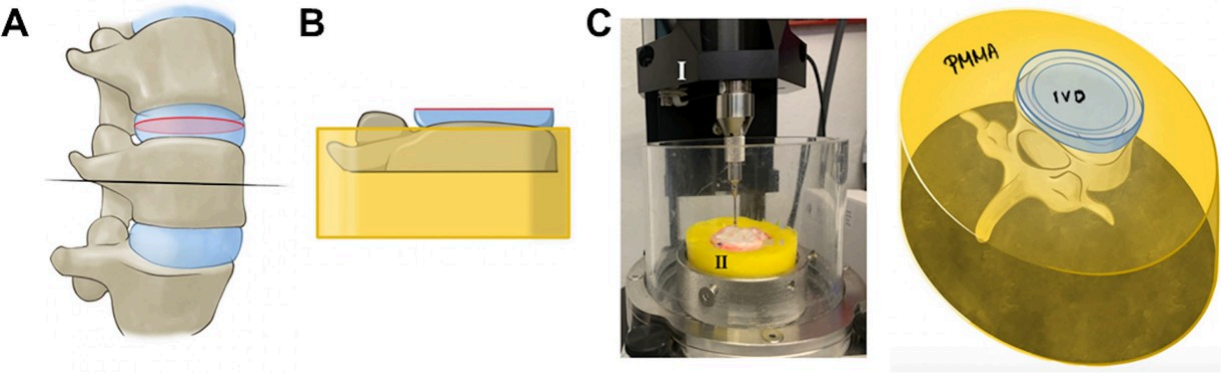
Biomechanical testing unit preparation for indentation analysis. A) Schematic representation of a spine segment. The black line indicates the cut made on the vertebral body. The red line indicates the cut made on the middle area of the intervertebral disc. B) Biomechanical testing unit composed of half intervertebral disc with its half vertebral body. C) Biomechanical indentation device (I). The biomechanical testing unit presented in (B) was mounted on bone cement to secure stability during testing (II).

Horizontally cut, half vertebral body and the attached half intervertebral disc (Fig 4A) was mounted in bone cement (Fig 4: 2B and 2C, “casted-specimens”) and placed in the indentation-device cast as shown in Fig 4: 2C. Using a customized holder, the specimen was embedded in polymethylmethacrylate (PMMA, Technovit 3040, Kulzer GmbH, Germany). After the initial hardening procedure of the PMMA, the specimen was pressed into the PMMA, which had a rubber-like consistency. As the PMMA hardening is exothermal generating temperatures >70°C, the specimen had to be removed from the PMMA cast before the final hardening started to avoid any heat-related impact on the IVD. This process facilitated the creation of a bone-tailored cast with complete circumferential confinement of the specimen.

A total of 6 discs were isolated from 4 different caudal spine specimens.

### Automated indentation mapping

The “casted-specimens” were submerged into 8 mM RF-DMEM solution for 15 min in a dark environment (to avoid unintended RF activation from natural light sources) and placed on a specimen holder fitted onto a specimen chamber, installed on a multiaxis materials testing machine. (Mach-1, Biomomentum Inc., Canada). For the biomechanical mapping, IVD was macroscopically divided into the annulus fibrosus (AF) and nucleus pulposus (NP) areas and perpendicular indentation mapping was achieved by simultaneously moving the three stages of the testing machine [21]. In this way, a spherical indenter (diameter 2mm) was indented by 2mm at a speed of 0.5mm/s (Fig 4: 4C). These parameters were identified during pretests on identical specimens. Initially, a nondestructive indentation test utilizing the same spherical indenter was used to determine the relaxation behavior of the IVD before and after treatment with 8 mM RF followed by exposure to 3.5 mW/cm^2^ UVA light (RF+UVA). Samples were aligned again as before on the device and a second indentation-mapping was performed utilizing the same coordinates, thus facilitating a direct comparison of the mechanical properties before and after the UVA-RF induced crosslinking. Lastly, the thickness of the IVD was identified, using a needle technique, at the same test locations as during the indentation tests and the resultant instantaneous modulus (IM) was calculated [22, 23]. Triplicate sets of data were collected from each measured region.

### Statistical analysis

A statistical software package (Graphpad Prism 7.0, San Diego, CA) was used to conduct the analyses. Results are presented in bar plots as median ± interquartile range (IQR). Normality was assessed using Shapiro-Wilk normality test. For normally distributed data, the differences were assessed using two-way ANOVA. Non-parametric Kruskal-Wallis test followed by Dunn’s multiple comparison test was used to analyze non-parametric data. The data acquired from the automated indentation mapping were non-normally distributed; therefore, non-parametric Wilcoxon testing was performed to compare the indentation results before and after UVA-CXL. *P-values* < 0.05 were considered significant.

## Results

### Viability of AF cells immediately after treatment

**Fig 5:**
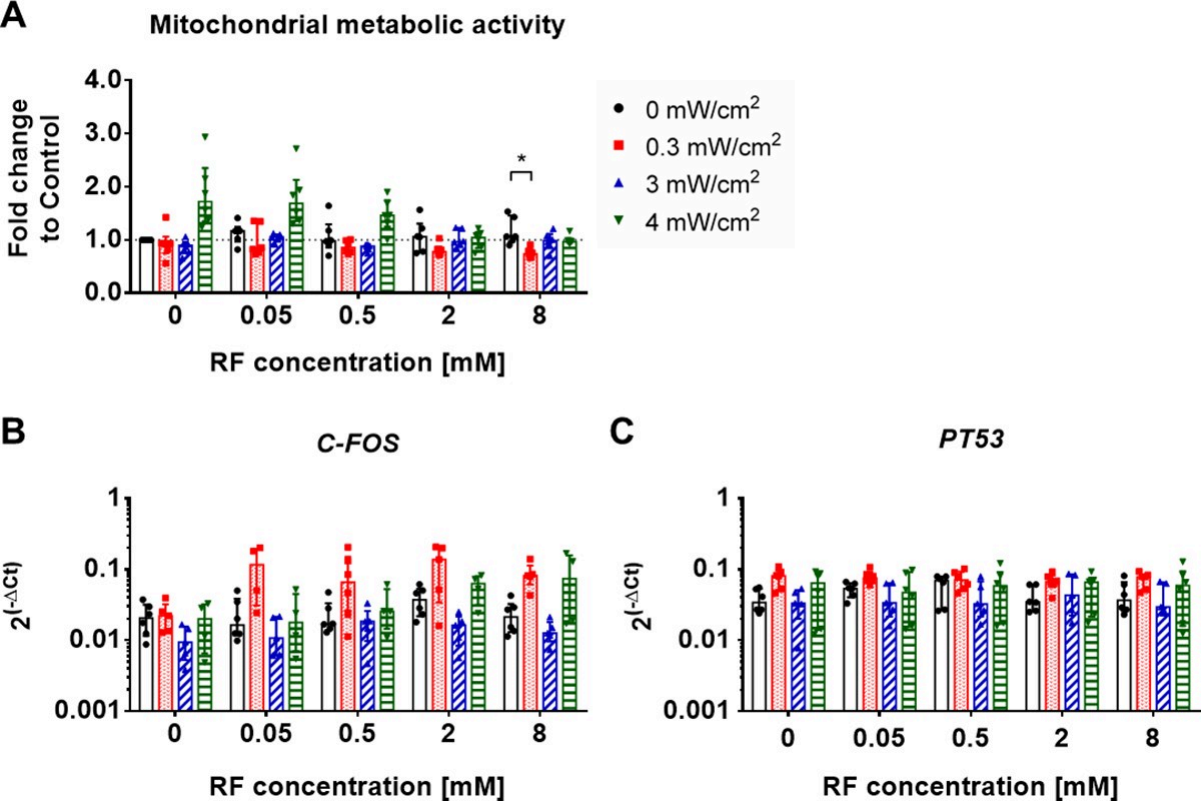
Viability analysis of AF cells directly after Riboflavin (RF) and UVA light treatment. A) Mitochondrial metabolic activity normalized to the DNA amount and the untreated control group (DMEM alone; control = 1, dashed line). B) mRNA expression of bovine *C-FOS* and *PT53* normalized to *GAPDH* housekeeping gene. Results are presented as median ± IQR (n = 4–6; 2 biological replicates and 2–3 experimental replicates). Kruskal-Wallis test was used for statistical analysis, *P < 0.05.

The metabolic activity of AF cells was assessed by means of the Resazurin assay and the relative fluorescence units were normalized to DNA content and to the untreated control group (Fig 5A). The combined treatment of 8 mM RF and 0.3 mW/cm^2^ UVA irradiation significantly decreased the metabolic activity of AF cells in contrast to the 8 mM RF treatment alone (*P<0.05). Although without significant differences, UVA irradiation of AF cells with 4 mW/cm^2^ alone, as well as in combination with 0.05- or 0.5 mM RF tended to increase the metabolic activity of AF cells (by 1.8±0.6-, 1.8±0.5- and 1.4±0.3-fold, respectively), immediately after treatment compared to the untreated control group. Gene expression analyses did not show significant differences in the expression of the metabolic activity marker *C-FOS* (Fig 5B) or the apoptosis regulator *PT53* (Fig 5C) between the analyzed groups. Although no statistically significant differences were observed for *C-FOS* expression, a consistent trend was noticed with 4.5-fold increase, comparing the treatment with 0.05–8 mM RF and 0.3 mW/cm^2^ UVA and the treatments with 0.3 mW/cm^2^ UVA alone or RF alone (Fig 5B).

### Viability of AF cells 24 hours after treatment

**Fig 6:**
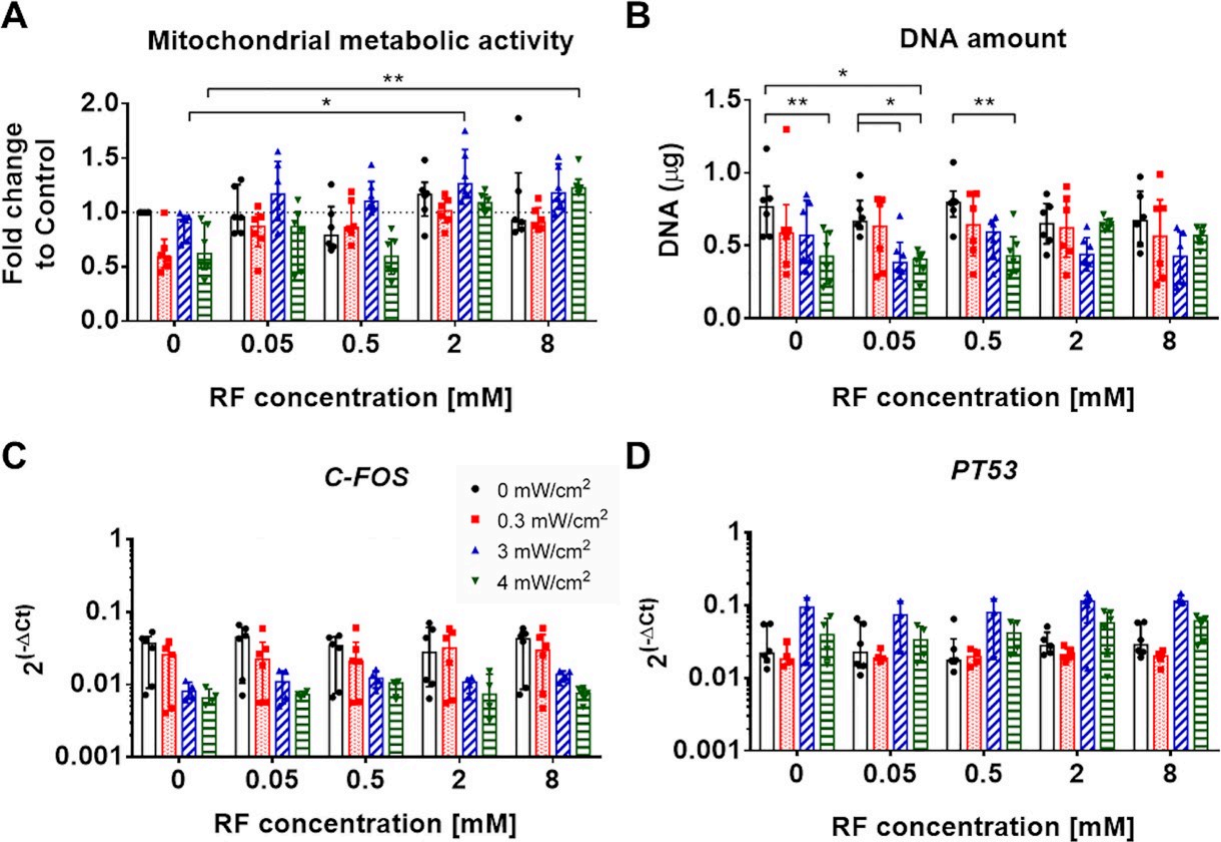
Viability analysis of AF cells 24 hours after Riboflavin (RF) and UVA light treatment. A) Mitochondrial metabolic activity normalized to the DNA amount and the untreated control group (DMEM alone; control = 1, dashed line). B) mRNA expression of bovine *C-FOS* and *PT53* normalized to *GAPDH* housekeeping gene. Results are presented as median ± IQR (n = 4–6; 2 biological replicates and 2–3 experimental replicates). Kruskal-Wallis test was used for statistical analysis of mitochondrial metabolic activity and *C-FOS* and *PT53* gene expression, whereas two-way ANOVA was used for the statistical analysis of DNA amount, *,P < 0.05, **,P < 0.01.

Twenty-four hours after treatment, no differences in the metabolic activity were observed between the AF treated cells and untreated control group Fig 6A). However, significantly higher metabolic activity was found in both AF cells treated with 2 mM RF and 3 mW/cm^2^ UVA and with 8 mM RF and 4 mW/cm^2^ UVA, in contrast to the 3 mW/cm^2^ (*, P<0.05) or 4 mW/cm^2^ (**, P<0.01) UVA irradiation only. DNA expression was significantly lower in samples treated with 4 mW/cm^2^ UVA only (**, P<0.01), or with 0.05 mM RF and 4 mW/cm^2^ UVA (*, P<0.05) in contrast to untreated controls (Fig 6B). The groups treated with 0.05 mM RF and 3 or 4 mW/cm^2^ also presented lower DNA content, compared to the 0.05 mM RF treatment only. Treatment with 0.5 mM RF and 4 mW/cm^2^ UVA also led to lower DNA content than treatment with 0.5 mM RF only (**, P<0.01). *C-FOS* and *PT53* expression was not altered by the different treatment modalities (Fig 6C and 6D).

Possible DNA fragmentation in AF apoptotic cells was investigated using the CFTM 488A TUNEL dye (green). Representative images of AF cells, 24 hours after treatment with 0–8 mM RF plus 4 mW/cm^2^ UVA, are depicted in Fig 2. A positive apoptosis control (treatment with 75% DMSO) was included. Every condition was analyzed in triplicate. Overall, no apoptotic cells were detected in any of the RF- and/or UVA-treated groups, neither immediately (data not shown) nor 24 hours after treatment (Fig 2) the cells remained homogeneously distributed on the plates. The positive control (treatment with 75% DMSO) displayed apoptotic AF cells, thus confirming that the assay was working.

### Delamination strength of fresh AF tissue

The delamination strength of the adjacent AF lamellae was significantly higher after UVA-CXL treatment compared to the untreated control (*, P<0.05; Fig 3), whereas treatment with RF or UVA alone did not show any changes in delamination strength compare to the control group [20].

### Indentation mapping of IVD

**Fig 7:**
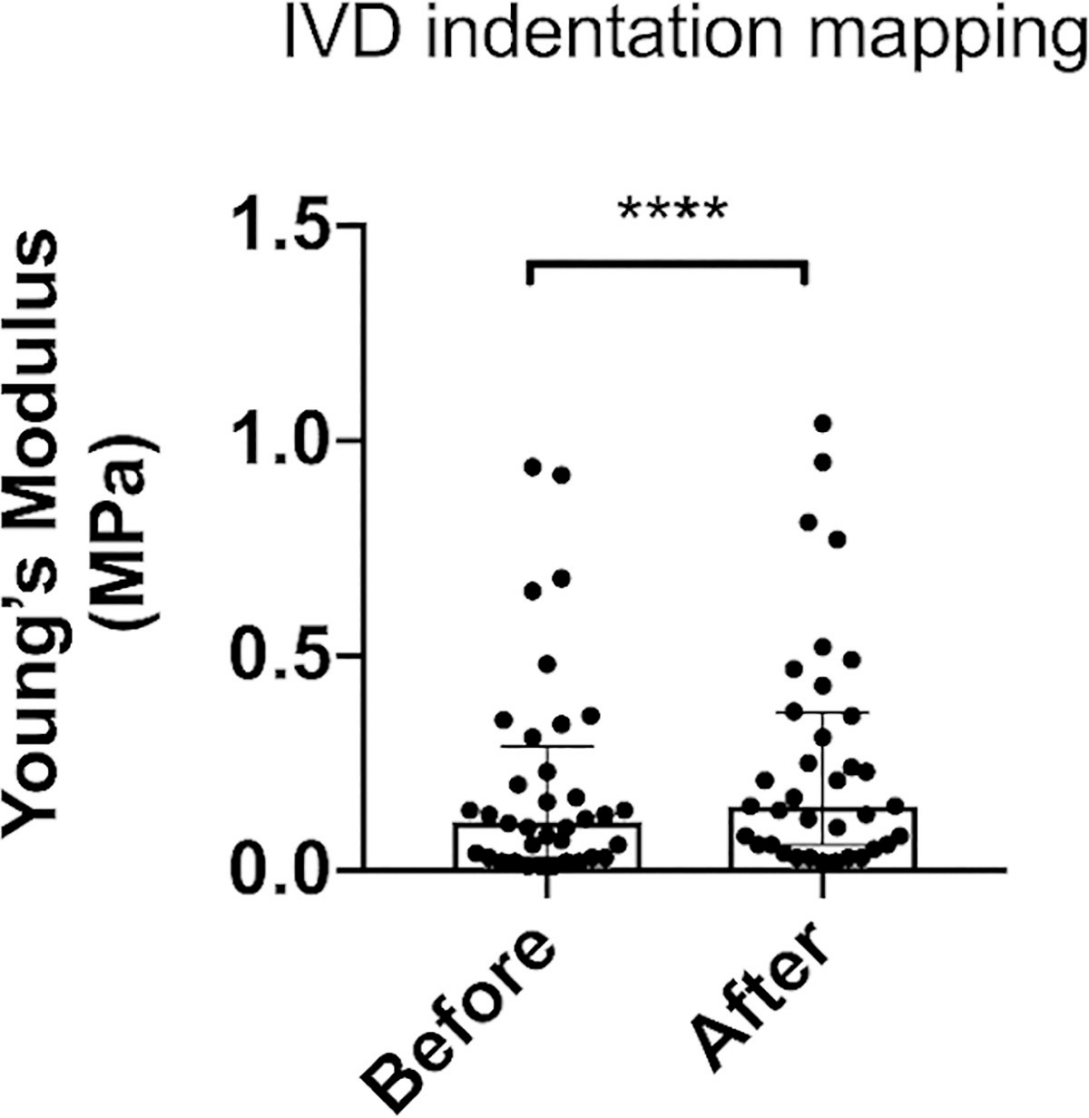
Automated indentation mapping of IVD tissue. The relaxation behavior of the IVD tissue was determined before and after treatment of IVD tissues with 8 mM Riboflavin followed by exposure to 3.5 mW/cm^2^ UVA light (RF+UVA). A total of 6 “casted-specimens” isolated from n = 4 different caudal spine specimens, were analyzed.

The indentation-mapping analysis revealed that the UVA-CXL treatment significantly increased the instantaneous modulus (152.84±20.80%) of the healthy IVD tissue (Fig 7: ****, P<0.0001). Further analysis will follow in the discussion session.

## Discussion

Back pain, the worldwide single leading cause of disability, is mostly associated with overloaded or degenerated IVDs [1]. A major compensatory physiological response is endogenous cross-linking [6]. After a certain degree of degeneration, the ratio of newly built versus broken cross-links decreases, leading to irreversible tissue damage. Exogenous crosslinking counteracts the progression of tissue damage by supporting the physiological endogenous crosslinking response. It aims to reinforce the weakened or damaged IVD matrix in order to improve its mechanical loading resistance. To our knowledge, this is the first study, successfully introducing the ophthalmologically applied UVA-CXL in IVDs. Clinically applied RF and UVA parameters were well tolerated from IVD-isolated cells and testing prior and after UVA-CXL on healthy IVDs, revealed a significant biomechanical reinforcement effect.

Genipin, a natural exogenous crosslinking agent, showed in various studies promising results in reinforcing the IVD, but its efficiency depends on long intradiscal-incubation time (days) and requires an anatomically- intact IVD, in order for genipin to adequately diffuse as many as possible IVD layers [9, 10, 24, 25]. In contrary UVA-CXL has the advantage of an immediate and spatially targeted crosslinking effect, thus opening the way for intraoperative applications especially on IVDs already undergone irreversible tissue damage. Additionally, we have recently introduced novel nondestructive real-time assessment tools that can quantify and verify the UVA-CXL effect on IVD tissues further supporting our efforts to translate this effective ophthalmological intervention in spinal surgery [26].

The first part of this study evaluated IVD-cell responses induced after various RF and UVA parameters that are already clinically applied in ophthalmology. We used AF cells isolated from intact bovine discs displaying a spindle-shaped morphology [27]. Cells were expanded and exposed to treatment with RF, UVA, or combining both. Direct and delayed cellular responses (viability, metabolic activity, and gene expression) have been evaluated at two timepoints, immediately and 24 hours after the treatment.

The investigated parameters had none or a moderate effect on the metabolic cellular response. Regardless of the timepoint, both highest RF (8 mM) and UVA (4 mW/cm^2^) parameters were non-cytotoxic.

The reduction of cell viability after UVA treatment and their increment after RF treatment, has been already demonstrated by Schultz et al. in vaginal connective tissue cells [28]. Schultz et al. tried to clarify viability, apoptosis and necrosis of vaginal cells treated by RF and UVA-based photopolymerization in order to strengthen vaginal tissues at risk of prolapse. To achieve this goal, vaginal cells were exposed to different concentrations of RF (0.1 and 10%) and control medium, followed by UVA irradiation (3 mW/cm^2^). They showed that RF attenuated UVA-related phototoxicity and damage by inhibiting necrosis [28].

The influence of RF and UVA treatment on the expression of apoptosis regulator *TP53* and of cell-metabolic activity marker *C-FOS* by AF cells was also evaluated [29]. *TP53* is known to play a major role in cell proliferation, aging processes, and cell-death [30]. It is also known to respond to DNA damage or shortening of the telomere [31]. Simulated weightlessness using the tail-suspension method for a period of 8 weeks demonstrated on mice, that the expression of *TP53* is increased in degenerative IVDs [31]. In the present study UVA light irradiation caused downregulation of TP53 at every condition after 24 hours. However, *TP53* expression after RT-PCR analysis did not show any significant difference for any of the applied UVA light intensities or RF concentrations compared to the control cells. *C-FOS* is known to be involved in the expression of inflammatory cytokines and MMPs, which are associated with IVD degeneration [32, 33]. Overall, in our experiments, no detrimental effect on cell proliferation or apoptosis could be observed in AF cell cultures after the application of different RF and UVA treatment combinations up to 24 hours.

Additionally, we assessed cell viability by microscopic evaluation using the TUNEL assay. Our results clearly show that cells remain viable with no apparent differences between the tested conditions indicating that neither the high UVA light treatment, the high RF concentrations nor the combined UVA-CXL treatment led to a significant loss of cell viability. However, studies have shown in histologic evaluations of rabbit corneas after UVA-CXL a pattern of extensive keratocyte loss throughout the stroma, with concomitant endothelial loss [34]. In future experiments, we also plan to evaluate our samples histologically.

The second part of this study focused on the biomechanical effects of UVA-CXL on IVDs.

Gregory et al. noted that particularly the trans-lamellar bridging network between the AF lamellae, containing type VI collagen fibers proteoglycans and elastin, plays a major role in ensuring the matrix integrity [20, 35]. Resistance to delamination and the ability to contain the nucleus pulposus within the IVD is essential, since a reduction in peel strength was observed in degenerated IVD tissue. The mechanical AF-peel force test has been shown to provide a good method to test mechanical strength properties of AF tissue [18]. In our study, a significant increase in delamination strength after UVA-CXL was observed.

An automated indentation test was performed to evaluate UVA-CXL induced changes in the IVD-instantaneous modulus. Treating the IVD with the RF solution led as expected to tissue swelling. To eliminate this factor during our biomechanical profiling, we compared the instantaneous modulus on precisely mapped IVD areas after UVA-CXL with the same point after treatment with only RF solution. All measurements were taken in a dark environment to prevent unintended crosslinking. This method has shown a significant increment of the IVD modulus of about 153%. This is in line with previous literature findings in the ophthalmological field, showing a significant increment of the cornea rigidity after cross-linking (up to 300%) with long-lasting results (over 10 years) in patients with keratoconus [12, 16, 17]. Although we applied UVA-CXL on healthy bovine discs and utilized clinical parameters tailored for the sensitive corneal tissue, our results indicate a significant biomechanical enhancement of the IVD, supporting our hypothesis that UVA-CXL could be translated for spinal applications.

A major limitation of our study is the ex-vivo nature of our experimental setting. Our in vitro results have proven the feasibility and safety of this method for the disc cells and the immediate UVA-CXL effects on the IVDs-extracellular matrix, and thus set the stage for future experiments to answer concerns about the efficacy and long-term safety of the procedure. Another limitation was the uncontrolled diffusion of the RF solutions in the IVD samples. Future experiments will focus on quantifying how various RF formulations diffuse into the specific IVD regions. Additionally, this study could not quantify the efficiency of UVA-CXL in different depths of the IVD-samples. The ophthalmological community has already identified the physical limitations of UVA irradiation as a RF activator and explores promising alternatives of non-linear near infrared laser activation for more focused and efficient crosslinking [36, 37]. We will address these questions in future experiments. Finally, the experiments were performed using healthy cells and tissues. In the future, it will be of clinical relevance to evaluate how UVA-CXL performs in already degenerated IVD samples as well as in aged cell populations.

### In conclusion

This feasibility study showed safety in translating an ophthalmological UVA-CXL treatment approach on IVD tissues. IVD-isolated cells tolerated all UVA-CXL parameters and biomechanical testing revealed significant reinforcing effects on healthy tissue samples. This supports our hypothesis that UVA-CXL could reinforce the mechanical properties of IVD matrix with structural damage due to degenerative alterations and improve its functionality.
